# A Hyaluronan-Based Scaffold for the *in Vitro* Construction of Dental Pulp-Like Tissue

**DOI:** 10.3390/ijms16034666

**Published:** 2015-03-02

**Authors:** Letizia Ferroni, Chiara Gardin, Stefano Sivolella, Giulia Brunello, Mario Berengo, Adriano Piattelli, Eriberto Bressan, Barbara Zavan

**Affiliations:** 1 Department of Biomedical Sciences, University of Padova, Viale Giuseppe Colombo, 3, 35131 Padova, Italy; 2 Department of Neurosciences, University of Padova, Via Giustiniani, 2, 35131 Padova, Italy; 3 Department of Stomatology and Biotechnologies, University of Chieti-Pescara, Via dei Vestini, 31, 66100 Chieti, Italy

**Keywords:** hyaluronan, stem cells, human dental pulp stem cells (DPSCs), dental pulp, tissue engineering, regenerative endodontics

## Abstract

Dental pulp tissue supports the vitality of the tooth, but it is particularly vulnerable to external insults, such as mechanical trauma, chemical irritation or microbial invasion, which can lead to tissue necrosis. In the present work, we present an endodontic regeneration method based on the use of a tridimensional (3D) hyaluronan scaffold and human dental pulp stem cells (DPSCs) to produce a functional dental pulp-like tissue *in vitro*. An enriched population of DPSCs was seeded onto hyaluronan-based non-woven meshes in the presence of differentiation factors to induce the commitment of stem cells to neuronal, glial, endothelial and osteogenic phenotypes. *In vitro* experiments, among which were gene expression profiling and immunofluorescence (IF) staining, proved the commitment of DPSCs to the main components of dental pulp tissue. In particular, the hyaluronan-DPSCs construct showed a dental pulp-like morphology consisting of several specialized cells growing inside the hyaluronan fibers. Furthermore, these constructs were implanted into rat calvarial critical-size defects. Histological analyses and gene expression profiling performed on hyaluronan-DPSCs grafts showed the regeneration of osteodentin-like tissue. Altogether, these data suggest the regenerative potential of the hyaluronan-DPSC engineered tissue.

## 1. Introduction

Dental pulp is a loose connective tissue in the central cavity of each tooth composed of four layers of cells: the external odontoblastic layer, the cell-free zone, the cell-rich zone and the internal parietal plexus of nerves. Deep inside, there is a great amount of fibroblasts and extracellular matrix (ECM). Other cells in the pulp include undifferentiated mesenchymal cells, fibrocytes, macrophages and lymphocytes. Dental pulp also contains blood vessels and nerves, which enter in the pulp cavity through the apical foramen [1].

The primary function of dental pulp tissue is to maintain the vitality of the tooth and to produce dentin. However, the pulp tissue is particularly vulnerable to external insult, because the small volume of tissue is contained in an unyielding dentin casing, its only available blood supply coming from one end, at the root apex [2].

Dental pulp disease typically progresses in several stages, described in various classifications as clinically normal pulp, pulpitis (which may or may not be reversible) and necrosis [3,4].

Directly capping dental pulp with various cement-based compounds containing hyaluronic acid (HA), calcium hydroxide or mineral trioxide aggregate, for instance, promotes dentinogenesis [5,6,7,8]. Cells remaining within the healthy portion of the pulp migrate to the injured site, proliferate in response to the growth factors released from the surrounding dentin matrix and become attached to the necrotic layer to form osteodentin. The cells attached to the osteodentin subsequently differentiate into odontoblasts, forming a reparative dentin that preserves the pulp’s integrity [9].

Dental pulp stem cells (DPSCs) play an important part in both oral and non-oral regenerative medicine because of their biological properties, which include multipotency, high proliferation rates and accessibility [10,11,12,13]. In addition, Karaöz *et al.* demonstrated that DPSCs are able to differentiate into both neural and vascular endothelial cells *in vitro* [14]. An ideal type of therapy might involve regenerative approaches in which diseased or necrotic pulp tissues are removed and replaced with regenerated pulp tissues to revitalize the teeth. In particular, regenerative pulp therapy would re-establish the normal tissue continuum at the pulp-dentin border by regulating the tissue-specific processes of reparative dentinogenesis [15,16]. One of the key aspects of this modern strategy concerns the use of biodegradable materials as scaffolds for the tridimensional (3D) growth of the cells previously expanded *in vitro*. The scaffolds provide a 3D environment for cells to attach and grow, therefore mimicking the *in vivo* condition. Apart from being biodegradable, an ideal scaffold should be biocompatible and have adequate physical and mechanical strength. Then, it should be porous to allow the placement of cells and effective transport of nutrients, oxygen, waste, as well as growth factors. Finally, it should be replaced by regenerative tissue, while retaining the shape and form of the final tissue structure [1]. Various natural and synthetic biomaterials have been investigated as scaffolds for dental pulp tissue regeneration, such as ECM components, self-assembling systems, hydrogels or bioceramics [17,18,19,20,21,22].

A promising biomaterial in the field of tissue engineering and regenerative medicine is HA (also called hyaluronan) and its derivatives, obtained by HA esterification with different alcohols. The resulting hyaluronan-based biodegradable polymers have been used successfully with bone marrow- and adipose-derived mesenchymal stem cells in *in vitro* and *in vivo* studies [23,24,25] and in clinical applications [26,27]. Hyaluronan is a ubiquitous molecule of connective tissues. It has a fundamental role in many biological processes, such as water balance, cell recognition, embryonic development and wound healing, both in adult and fetal life stages. Compared to the above-mentioned materials, hyaluronan has more structural and physiological functions within tissues, including extracellular and cellular interactions, growth factor interaction and regulation of osmotic pressure and tissue lubrication, which help to maintain the structural and homeostatic integrity of tissues. Moreover, hyaluronan is quickly turned over in the body by hyaluronidase, with tissue half-lives ranging from hours to days [28].

Focusing on dentin-pulp complex regeneration, a hyaluronan-based scaffold can be projected to adapt easily to the variable shape of the pulp chamber, to have a relatively fast setting time and to prove amenable to root canal modeling and adaptation. The dental pulp tissue expresses hyaluronan in a different manner during tooth development; hyaluronan contributes to the development of the dentine matrix and dental pulp growth, but during odontogenesis, its expression gradually decreases with age [29]. The role of hyaluronan in preserving dental pulp vitality and inducing dentin repair has already been investigated. *In vivo* experiments suggest that hyaluronan alone or in combination with other biomaterials could provide a suitable environment for reparative dentin induction through mesenchymal stem cells differentiation [30,31,32,33].

In the present study, we propose a method for the *in vitro* reconstruction of a dental pulp-like tissue that combines the numerous properties of hyaluronan with the stemness of DPSCs. A population of DPSCs was isolated from third molars of healthy patients and seeded onto hyaluronan-based non-woven meshes in the presence of a sequential cocktail of differentiation factors, including neuronal, glial, endothelial and osteogenic factors. We have evaluated the *in vitro* neuronal, glial, endothelial and osteogenic commitment of DPSCs on 3D scaffolds by gene expression profiling and immunofluorescence (IF) analyses. Furthermore, we have filled the artificial construct in critical-size bone defects to assess the osteodentin-like tissue regeneration *in vivo*.

## 2. Results and Discussion

### 2.1. DPSC Isolation and Amplification

**Figure 1:**
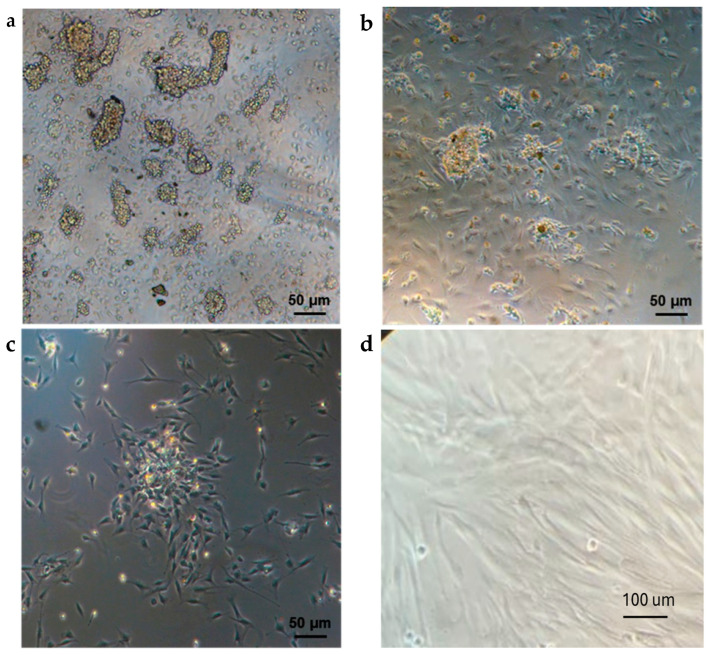
Morphological features of dental pulp stem cells (DPSCs). (**a**) One day after seeding; (**b**) After three days from seeding; (**c**) After the first passaging step; (**d**) DPSCs at Passage 2 (p2).

An enriched population of DPSCs was isolated from third molars of healthy human subjects and expanded on tissue culture plates in the presence of basal medium. One day after seeding, the adherent DPSCs showed a round morphology and the ability to form colonies of different sizes (Figure 1a). After three days from seeding, the morphology of DPSCs was heterogeneous and varied among cuboidal and spindle-shaped (Figure 1b). After the first passaging step (Passage 1, p1), most cells appeared spindle-shaped, but cell colonies were still present (Figure 1c). After two passages (p2), DPSCs acquired their final morphology: spindle-shaped and fibroblast-like (Figure 1d). Overall, the expanded DPSCs revealed a good proliferative potential and the ability to achieve confluence quickly.

### 2.2. In Vitro Construction of a Dental Pulp-Like Tissue

In a previous study published by our group [34], the stemness of the isolated cells has been evaluated by analyzing their potential to differentiate into cells of mesodermal origin, like adipocytes, chondrocytes or osteocytes. When cultured in basal medium, DPSCs expressed the stem cell marker S100. On the contrary, in presence of differentiation media, the cells showed increased expression of the specific differentiation markers, such as osteocalcin, osteonectin, osteopontin and Von Willebrand factor, CD31 for osteogenic and endothelial commitment, respectively [34]. In addition, these cells have also been able to acquire a neuronal- or glial-like phenotype when properly stimulated. For example, the expression of beta-III tubulin was clearly detectable when DPSCs were cultured in neuronal differentiation medium, whereas the GFAP and CNPase mRNAs were found upregulated in the presence of glial differentiation medium [34].

**Figure 2:**
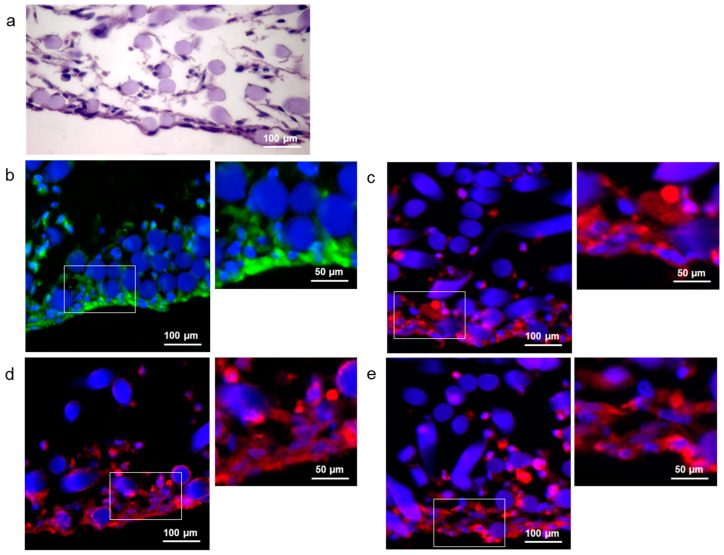
DPSCs seeded onto hyaluronan-based scaffolds and cultivated with differentiation medium for 21 days. (**a**) H/E staining; Fluorescence images of cells expressing (**b**) collagen type I (green), a component of the extracellular matrix (ECM); (**c**) Von Willebrand factor (red), an endothelial marker; (**d**) Beta-III Tubulin (red), a neuronal marker; and (**e**) GFAP (red), a glial marker. Cell nuclei and the biomaterial are stained blue.

Based on these interesting results, we proceeded with the development of a dental pulp-like tissue *in vitro*. As described above, dental pulp tissue contains different cell types: odontoblasts, endothelial cells, neurons and glial cells. In order to regenerate this complex structure, DPSCs were seeded onto hyaluronan-based scaffolds previously embedded with neuronal and glial factors, then cultivated with osteo-endothelial differentiation medium for 21 days. Hematoxylin/eosin (H/E) staining showed that the cells were distributed both on the surface of the matrix and within the 3D construct. The DPSCs grew well inside the hyaluronan-based scaffold, filling all of the spaces between the fibers (Figure 2a).

In order to assess the ECM production by DPSCs, IF staining of collagen type I, the major fibrous component of the dental pulp ECM, was performed. Positive staining for collagen type I (Figure 2b, in green) confirmed the ability of DPSCs to synthesize ECM components within hyaluronan fibers. After 21 days of 3D *in vitro* culture, the DPSCs showed the acquisition of endothelial, neuronal and glial phenotypes. Furthermore, in this case, the commitment and the 3D organization of the differentiated cells was evaluated by IF staining. The evidence of an endothelial phenotype was revealed by the presence of Von Willebrand factor (Figure 2c, in red), whereas neuronal and glial commitment was detected by the presence of beta-III tubulin and glial fibrillary acidic protein (GFAP) markers, respectively (Figure 2d,e, in red). In all figures, blue represents both cell nuclei and the hyaluronan-based biomaterial.

**Figure 3:**
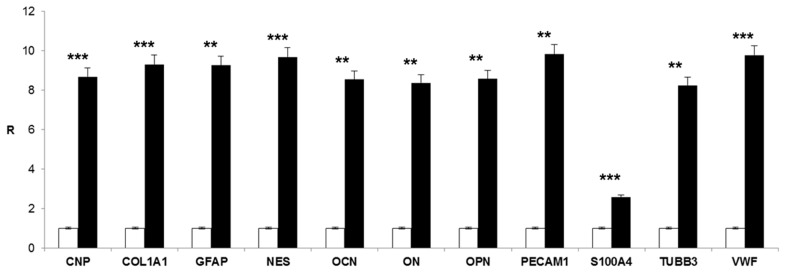
Gene expression profile of endothelial markers (Von Willebrand factor (VWF), platelet/endothelial cell adhesion molecule 1 (PECAM1)), osteo/odontoblast-related markers (osteocalcin (OCN), osteonectin (ON), osteopontin (OPN), collagen type I (COL1A1)), glial cell markers (glial fibrillary acidic protein (GFAP), 2',3'-cyclic nucleotide 3' phosphodiesterase (CNP)), neuronal markers (nestin (NES), tubulin beta 3 class III (TUBB3)) and stem cell marker S100A4 in DPSCs seeded onto hyaluronan-based scaffolds and cultivated with differentiation medium (black bars) or with basal medium (white bars) for 21 days. Significant differences between DPSCs in differentiation medium and DPSCs in basal medium (** *p* < 0.01; *** *p* < 0.001); R: relative expression.

At the same time, gene expression analysis of multiple differentiation markers was performed. The expression of endothelial, osteo/odontogenic, neuronal and glial markers was evaluated after 21 days in DPSCs seeded onto hyaluronan-based scaffolds in the presence of the differentiation medium with respect to the 3D constructs cultivated in basal medium. As shown in Figure 3, high levels of expression were detected for Von Willebrand factor (VWF) and platelet/endothelial cell adhesion molecule 1 (PECAM1), two markers specific of endothelial cells. Significant expression levels were also measured for the osteo/odontoblast-related markers, such as osteocalcin (OCN), osteonectin (ON), osteopontin (OPN), as well as for collagen type I (COL1A1). There was also specific expression of glial cell markers, such as GFAP and 2',3'-cyclic nucleotide 3' phosphodiesterase (CNP). Furthermore, the differentiated cells expressed nestin (NES) and tubulin beta 3 class III (TUBB3), which suggested a neurogenic potential of DPSCs. On the other hand, the low expression of the stem cell marker S100 calcium binding protein A4 (S100A4) confirmed the acquisition of a differentiated phenotype by DPSCs.

Taken together, these results seem to demonstrate that DPSCs were able to acquire all of the main phenotypes of the cells found in dental pulp tissue under specific culture conditions. These included the presence of the hyaluronan matrix treated with neural factors, as well as the addition of osteo-endothelial induction factors in the culture medium.

### 2.3. In Vivo Experiments

In order to evaluate the bone regenerative properties of the hyaluronan-DPSCs construct, the dental pulp-like tissue obtained *in vitro* was used to repair a calvarial defect in the rat. Implanted hyaluronan scaffolds without cells were used as a control for assessing the influence of DPSCs in the process of bone regeneration *in vivo*. After one month, bone grafts were removed from the skull and used for histological and gene expression analyses.

**Figure 4:**
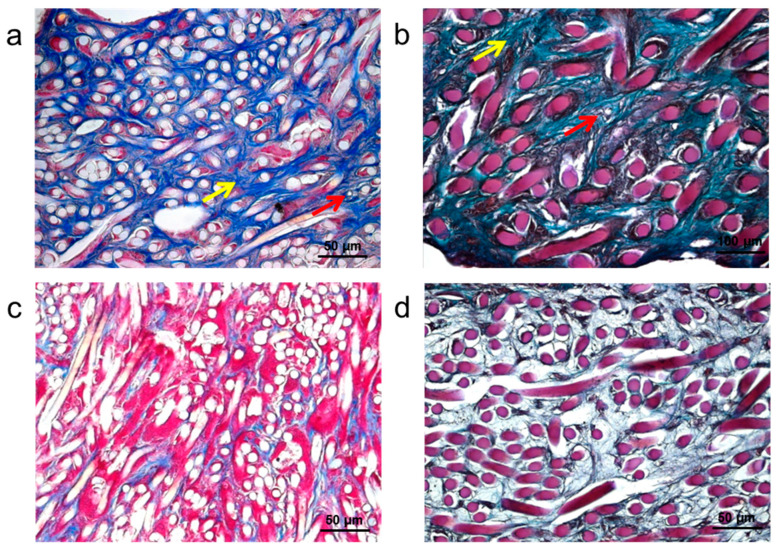

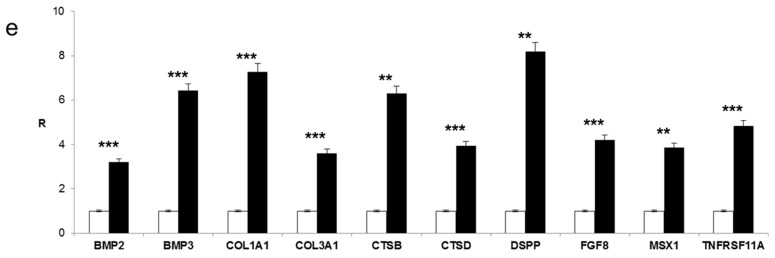
Histological analyses of hyaluronan-DPSCs bone graft one month after rat calvarial defect repair. (**a**) Azan Mallory staining of collagen matrix (yellow arrow), blood vessels (red arrow) and hyaluronan fibers (white circles); (**b**) Masson’s trichrome staining of collagen matrix (yellow arrow), blood vessels (red arrow) and hyaluronan fibers (purple circles); (**c**) Azan Mallory and (**d**) Masson trichrome staining of hyaluronan bone graft without cells; and (**e**) Gene expression profile of dentin ECM-related markers (dentin sialophosphoprotein (DSPP), bone morphogenetic protein 2 (BMP2), BMP3), bone ECM-related markers (COL1A1, COL3A1, cathepsin B (CTSB), cathepsin D (CTSD)) and other bone markers (fibroblast growth factor 8 (FGF8), msh homeobox 1 (MSX1), tumor necrosis factor receptor superfamily (TNFRSF11A)) in the hyaluronan-DPSC bone graft (black bars) compared to the hyaluronan scaffold alone (white bars). Significant differences between hyaluronan-DPSCs bone graft and hyaluronan scaffold alone (** *p* < 0.01; *** *p* < 0.001); R: relative expression.

As shown by the Azan Mallory (Figure 4a) and Masson trichrome stainings (Figure 4b), the tissue showed several signs of regeneration. As the hyaluronan fibers (white and purple circles, respectively) degraded, blood vessels (red arrows) and a collagen matrix (yellow arrows) became apparent throughout the tissue at the site of the perforation. No inflammatory cells were visible inside or outside the tissue. The outer portions of the perforation site showed cellular proliferation, indicating the progression of tissue regeneration in this area. On the contrary, the Azan Mallory (Figure 4c) and Masson trichrome stainings (Figure 4d) of the hyaluronan scaffold without cells do not show the presence of blood vessels and ECM components around the hyaluronan fibers. This observation could be related to the regenerative capabilities of the implanted DPSCs.

In order to verify this hypothesis, the expression of dentin and osteoblast differentiation-related markers was evaluated in the transplanted tissues by real-time PCR (Figure 4e). Specific gene expression was identified for dentin ECM, growth factors and proteins related to bone ECM. In particular, high levels of genes related to dentin ECM, such as dentin sialophosphoprotein (DSPP), bone morphogenetic protein 2 (BMP2) and BMP3, were observed in the cell-based HA scaffolds compared to the empty biomaterials. Furthermore, mRNA coding for proteins related to bone ECM, such as COL1A1, collagen type III (COL3A1), cathepsin B (CTSB) and cathepsin D (CTSD), was significantly expressed. Other bone markers, such as msh homeobox 1 (MSX1), an osteoproliferative transcription factor, the tumor necrosis factor receptor superfamily (TNFRSF11A) and fibroblast growth factor 8 (FGF8), were found upregulated in test samples with respect to control samples.

These results together demonstrate that the presence of DPSCs on the implanted scaffolds positively influenced the osteogenic regenerative process of the tissue.

## 3. Experimental Section

### 3.1. Biomaterial

The biomaterial used in the present study was derived from the total esterification of HA (synthesized from 80 to 200 kDa sodium hyaluronate) with benzyl alcohol and named HYAFF-11™ (Fidia Advanced Biomaterials, Abano Terme, Padova, Italy). The end product is a non-crosslinked linear polymer of unknown molecular weight that is insoluble in aqueous solution, but hydrolyzes spontaneously over time, releasing benzyl alcohol and hyaluronan.

HYAFF-11™ was used to create non-woven meshes (50 μm-thick fibers, specific weight of 100 g/m^2^). The properties of these substrates are described in detail elsewhere [25].

### 3.2. DPSCs Isolation

Human dental pulp samples were extracted from healthy third molars removed for orthodontic purposes from 18- to 35-year-old adults, after obtaining their informed consent. The Ethical Committee of Padua Hospital approved the research protocol. Before extraction, all individuals were checked for systemic and oral infections or diseases, and only disease-free subjects were selected for pulp collection. Each subject was pretreated for a week with professional dental hygiene. Before the extraction, the dental crown was coated with a 0.3% chlorhexidine gel (Forhans, New York, NY, USA) for 2 min. After mechanical fracturing, the dental pulp was harvested using a dental excavator or a Gracey curette. The pulp was gently removed and immersed for 1 h at 37 °C in a digestive solution containing 3 mg/mL collagenase type I from *Clostridium histolyticum* (Worthington Biochemical Corporation, Lakewood, NJ, USA) and 4 mg/mL Dispase II from *Bacillus polymyxa* (Roche Applied Science, Basel, Switzerland). After digestion, the solution was filtered through 70-µm Falcon strainers (BD Biosciences, Mississauga, ON, Canada). The cells were then cultured in basal medium made of Dulbecco’s Modified Eagle Medium (DMEM) (Lonza S.r.l., Milano, Italy) supplemented with 10% fetal bovine serum (FBS) (Bidachem S.p.A., Milano, Italy) and 1% penicillin/streptomycin (P/S) (EuroClone, Milan, Italy).

### 3.3. Culture Media Preparation

The neuronal differentiation medium consisted of NeuroBasal Medium (Gibco by Life Technologies, Paisley, UK) containing 1% FBS, 1% P/S, 2% b27 serum-free supplement (Gibco), 10 µg/mL human recombinant beta nerve growth factor (bNGF) (ProSpec, East Brunswick, NJ, USA), 100 ng/mL human recombinant brain-derived neurotrophic factor (BDNF) (ProSpec) and 20 ng/mL human recombinant neurotrophin-3 (NT3) (ProSpec).

Glial differentiation medium was made of NeuroBasal Medium containing 1% FBS, 1% P/S, 1% N2 supplement (Gibco), 4 μM forskolin (Merck Millipore, Darmstadt, Germany) and 10 ng/mL human recombinant heregulin-β (ProSpec).

Osteo-endothelial differentiation medium was made of basal medium supplemented with 10 mM β-glycerophosphate (Sigma-Aldrich, Saint Louis, MO, USA), 10 nM dexamethasone (Sigma-Aldrich), 1 mg/mL heparin (Seromed, Berlin, Germany), 10 ng/mL platelet-derived endothelial cell growth factor (PD-ECGF) (Calbiochem, San Diego, CA, USA) and 10 ng/mL basic fibroblast growth factor (bFGF) (ProSpec).

### 3.4. DPSCs Differentiation in 3D

Before seeding the cells, squares (1 cm × 1 cm) of non-woven meshes were cut and laid down on a 24-well culture plate using sterile forceps and scissors. The biomaterial was then embedded with both neuronal and glial differentiation media for 24 h at 37 °C in a humidified chamber containing 5% CO_2_. After completing this process, the non-woven squares were dehydrated under sterile conditions, and DPSCs were seeded at a density of 5 × 10^5^ cells/cm^2^ in the presence of osteo-endothelial differentiation medium for 21 days.

### 3.5. In Vitro Staining

The 3D scaffolds loaded with DPSCs were embedded in an OCT compound (Bio-Optica, Milan, Italy) and then cut into 7 μm thick-sections with cryostat (Leica CM1950, Leica, Milano, Italy). The frozen sections were placed onto positive-charged slides and then fixed in 4% paraformaldehyde solution in PBS for 10 min.

For H/E staining, the sections were stained with the nuclear dye, hematoxylin (Sigma-Aldrich), and the counterstain, eosin (Sigma-Aldrich).

For IF staining, the sections were incubated in 2% bovine serum albumin (BSA, Sigma-Aldrich) solution in PBS for 30 min at room temperature. The sections were then incubated with the primary antibodies in 2% BSA solution in a humidified chamber overnight at 4 °C. The following primary antibodies were used: rabbit polyclonal anti-human Von Willebrand factor antibody 1:100 (A0082; Dako, Milan, Italy); rabbit polyclonal anti-GFAP antibody 1:100 (AB5804; Millipore Corporation, Danvers, MA, USA); mouse monoclonal anti-collagen type I 1:100 (C2456; Sigma-Aldrich); chicken polyclonal anti-beta III tubulin antibody 1:100 (AB9354; Millipore Corporation). IF staining was performed with secondary antibodies: anti-rabbit IgG (H + L), DyLight 549 labeled (KPL, Gaithersburg, MD, USA); anti-mouse IgG, DyLight 488 labeled (KPL); or anti-chicken IgG (H + L), DyLight 549 labeled (KPL), diluted 1:1000 in 2% BSA for 1 h at room temperature. Nuclear staining was performed with 2 µg/mL Hoechst H33342 (Sigma-Aldrich) solution for 5 min. The sections were coverslipped with a drop of mounting medium.

### 3.6. Real-Time PCR

Total RNA was extracted with the RNeasy Mini Kit (Qiagen GmbH, Hilden, Germany), including DNase digestion with the RNase-Free DNase Set (Qiagen). The RNA quality and concentration of the samples were measured using the NanoDrop™ ND-1000 (Thermo Scientific, Waltham, MA, USA).

For the first-strand cDNA synthesis, 1000 ng of total RNA of each sample were reverse transcribed with M-MLV Reverse Transcriptase (Invitrogen, Carlsbad, CA, USA), following the manufacturer’s protocol.

Human and rat primers were selected for each target gene with Primer 3 software (Table 1 and Table 2). Real-time PCRs were carried out using the designed primers at a concentration of 300 nM and FastStart SYBR Green Master (Roche Diagnostics, Mannheim, Germany) on a Rotor-Gene 3000 (Corbett Research, Sydney, Australia). Thermal cycling conditions were as follows: 15 min denaturation at 95 °C; followed by 40 cycles of denaturation for 15 s at 95 °C; annealing for 30 s at 60 °C; and elongation for 20 s at 72 °C. Differences in gene expression were evaluated by the 2^∆∆*C*t^ method [35]. For *in vitro* experiments, DPSCs cultured for 21 days on the 3D scaffold in basal medium were used as control condition. For *in vivo* experiments, bone grafts derived from the hyaluronan scaffold alone were considered the control condition. In both cases, values were normalized to the expression of the glyceraldehyde-3-phosphate dehydrogenase (GAPDH) internal reference, whose abundance did not change under our experimental conditions.

**Table 1:** Human primer sequences.

Gene Symbol	Forward Primer (5'→3')	Reverse Primer (5'→3')	Product Length (bp)
*CNP*	AACCTTCCTGTCTTCATCCTTAGC	ATCACAAGCCAACACACTCATTG	137
*COL1A1*	TGAGCCAGCAGATCGAGA	ACCAGTCTCCATGTTGCAGA	178
*GAPDH*	TCAACAGCGACACCCAC	GGGTCTCTCTCTTCCTCTTGTG	203
*GFAP*	AGGAAGATTGAGTCGCTGGAG	CGCCATTGCCTCATACTGC	177
*NES*	TCAGAGGGAAGGAGATAGAGAGTC	AGCCAGAAACCATATGTCAAGAGA	171
*OCN*	GCAGCGAGGTAGTGAAGAGAC	AGCAGAGCGACACCCTA	193
*ON*	TGCATGTGTCTTAGTCTTAGTCACC	GCTAACTTAGTGCTTACAGGAACCA	183
*OPN*	TGGAAAGCGAGGAGTTGAATGG	GCTCATTGCTCTCATCATTGGC	192
*PECAM1*	TCCAGCCAACTTCACCATCC	TGGGAGAGCATTTCACATACGA	171
*S100A4*	AGGGTGACAAGTTCAAGCTCAA	GCAGGACAGGAAGACACAGTA	176
*TUBB3*	GCTCTACGACATCTGCTTCCG	GAAGGGCACCATGTTGACG	163
*VWF*	ACGTATGGTCTGTGTGGGATC	GACAAGACACTGCTCCTCCA	159

*CNP*, 2',3'-cyclic nucleotide 3' phosphodiesterase; *COL1A1*, collagen, type I, alpha 1; *GAPDH*, glyceraldehyde-3-phosphate dehydrogenase; *GFAP*, glial fibrillary acidic protein; *NES*, nestin; *OCN*, osteocalcin; *ON*, osteonectin; *OPN*, osteopontin; *PECAM1*, platelet/endothelial cell adhesion molecule 1; *S100A4*, S100 calcium binding protein A4; *TUBB3*, tubulin, beta 3 class III; *VWF*, von Willebrand factor.

**Table 2:** Rat primer sequences.

Gene Symbol	Forward Primer (5'→3')	Reverse Primer (5'→3')	Product Length (bp)
*BMP2*	TGCTCAGCTTCCATCACG	TTCCTGCATTTGTTCCCGAA	152
*BMP3*	GATAGCCACGTCAGAGAAGC	GCTTTCTCTCCTCCCACACTC	139
*COL1A1*	AAAGATGGCGAAGCTGGAG	GAAACCTCTCTCGCCTCTTG	210
*COL3A1*	AATGGTGACAGAGGAGAAACG	CCTCGATGTCCTTTGATGCC	149
*CTSB*	TGTGGAGGTGTCTGCTGAG	GGAGGGATGGTGTAGGGTA	168
*CTSD*	GTCGGTTCCATGTAAGTCAGA	ACTTGGCTGCGATGAATACG	101
*DSPP*	AGCAGGCAACATCACAACC	TGACAGAGTAGATGAGTGGAGTG	122
*FGF8*	CATGGCAGAAGACGGAGAC	CCTTTGCCGTTGCTCTTGG	147
*GAPDH*	GGCCTTCCGTGTTCCTA	AAGGTGGAAGAATGGGAGTTG	192
*MSX1*	GGGATGCAAAGGCGAAGA	GCTTTCTATAGGGCTGGGCTC	186
*TNFRSF11A*	CAACTCAACGGATGGCTACA	TGGCTGCTGCTTCACTG	134

*BMP2*, bone morphogenetic protein 2; *BMP3*, bone morphogenetic protein 3; *COL1A1*, collagen, type I, alpha 1; *COL3A1*, collagen, type III, alpha 1; *CTSB*, cathepsin B; *CTSD*, cathepsin D; *DSPP*, dentin sialophosphoprotein; *FGF8*, fibroblast growth factor 8; *GAPDH*, glyceraldehyde-3-phosphate dehydrogenase; *MSX1*, msh homeobox 1; *TNFRSF11A*, tumor necrosis factor receptor superfamily, member 11a, NF-κB activator.

### 3.7. In Vivo Experiments

The bone regenerative properties of hyaluronan-DPSCs constructs were evaluated by calvarial implantation in 8-week-old female nude rats (*n* = 3) (Wistar-NIH-FOXN1, Charles River). Two critical defects were generated for each rat’s skull: one was filled with hyaluronan scaffolds alone (control samples) and the other with hyaluronan-DPSCs construct (test samples). Test samples were prepared as those for the *in vitro* experiments and maintained in culture for 7 days before being implanted.

All surgical procedures were performed under general anesthesia with intraperitoneal injections of ketamine hydrochloride (Ketaras, Yuhan Corp., Seoul, Korea, 40 mg/kg) and xylazine (Rumpens Bayer Korea Ltd., Seoul, Korea, 10 mg/kg). The calvarial skin was disinfected with 10% betadine (Potadines, Sam-II Pharm., Seoul, Korea); then, 2% lidocaine containing 1:100,000 epinephrine (Lidocaine HCL, Epinephrine Inj., Yuhan Corp., Korea) was subcutaneously injected into the calvarial bone. At this point, an incision was made along the sagittal suture. The periosteum was raised, and a calvarial bone defect 5 mm in diameter was created using a trephine burr without perforating the dura. The area of the defect was filled with either the control or the test samples. All animals were treated and handled in accordance with the “Recommendations for Handling Laboratory Animals for Biomedical Research” compiled by the Committee on the Safe and Ethical Handling Regulations for Laboratory Experiments at the University of Padova, Padova, Italy. The animals were housed separately in temperature-controlled cages (22 °C) with a 12-h day/night cycle and were unrestrained, with food available “*ad libitum*”.

After 1 month, the animals were sacrificed under formalin perfusion, and the calvarial bone was removed from the skull.

For histological analyses, the calvarial bone was fixed in 4% paraformaldehyde solution in PBS overnight, decalcified with 10% EDTA (Sigma-Aldrich), pH 7.2, then paraffin-embedded and cut into 7 μm-thick sections. The bone graft sections were stained with Azan Mallory (Bio-Optica) or with Masson trichrome (Sigma-Aldrich).

For gene expression analyses, bone grafts were processed as described above (Section 3.6.).

### 3.8. Statistical Analysis

One-way analysis of variance (ANOVA) was used to analyze the data.

Levene’s test was used to demonstrate the equal variances of the variables. Repeated measures analysis of variance (Re-ANOVA), with a *post hoc* analysis using Bonferroni’s multiple comparison test, was used to identify any significant changes (*p* < 0.05). * *p* < 0.05; ** *p* < 0.01; *** *p* < 0.001.

Repeatability was calculated as the standard deviation of the difference between measurements. All tests were performed using the SPSS 16.0 software package (SPSS Inc., Chicago, IL, USA) (licensed to the University of Padova, Italy).

## 4. Conclusions

The dental pulp is a complex tissue, which includes different specialized cells, blood vessels and nerves. In the present study, we propose a method for the *in vitro* reconstruction of a dental pulp-like tissue using a hyaluronan matrix as the scaffold for the 3D differentiation of DPSCs towards neuronal, glial, endothelial and osteogenic phenotypes. Hyaluronan was chosen because it is a molecule of ECM that is distributed ubiquitously. Moreover, it is a biodegradable polymer that can be projected to adapt easily to the variable shape of the pulp chamber. On the other hand, DPSCs, the source of stem cells within the dental pulp, can differentiate into several cell lineages under defined conditions. In the present work, a population of DPSCs was seeded onto hyaluronan-based scaffolds in the presence of a sequential cocktail of differentiation factors, including neuronal, glial, endothelial and osteogenic factors. Gene expression profiling and immunofluorescence analyses confirmed the neuronal, glial, endothelial and osteogenic commitment of DPSCs on 3D scaffolds *in vitro*. Furthermore, the *in vivo* engraftment of hyaluronan scaffolds seeded with DPSCs in critical-size bone defects in rats confirmed the osteo-regenerative properties of these constructs.

In conclusion, these data demonstrate that the combination of a hyaluronan-based biomaterial and DPSCs could be a good strategy to generate a dental pulp-like tissue having the ability to repair a calvarial defect in an *in vivo* rat model.
