# Correction: Ferroni et al. A Hyaluronan-Based Scaffold for the *in Vitro* Construction of Dental Pulp-Like Tissue. *Int. J. Mol. Sci.* 2015, *16*, 4666–4681

**DOI:** 10.3390/ijms252111556

**Published:** 2024-10-28

**Authors:** Letizia Ferroni, Chiara Gardin, Stefano Sivolella, Giulia Brunello, Mario Berengo, Adriano Piattelli, Eriberto Bressan, Barbara Zavan

**Affiliations:** 1 Department of Biomedical Sciences, University of Padova, Viale Giuseppe Colombo, 3, 35131 Padova, Italy; letizia.ferroni@unipd.it (L.F.); chiara.gardin@unipd.it (C.G.); 2 Department of Neurosciences, University of Padova, Via Giustiniani, 2, 35131 Padova, Italy; stefano.sivolella@unipd.it (S.S.); giulia-bru@libero.it (G.B.); mario.berengo@unipd.it (M.B.); eriberto.bressan@unipd.it (E.B.); 3 Department of Stomatology and Biotechnologies, University of Chieti-Pescara, Via dei Vestini, 31, 66100 Chieti, Italy; a.piattelli@unich.it

In the original publication [1], there was a mistake in Figure 1d as published. An error occurred during the selection process of the cell images, leading to the inadvertent inclusion of an incorrect photograph. This issue arose due to a misalignment in the imaging analysis, resulting in the selection of the wrong visual data. We are currently addressing this mistake to ensure the accuracy of our materials. The corrected Figure 1d appears below. The authors state that the scientific conclusions are unaffected. This correction was approved by the Academic Editor. The original publication has also been updated. 

## Figures and Tables

**Figure 1:**
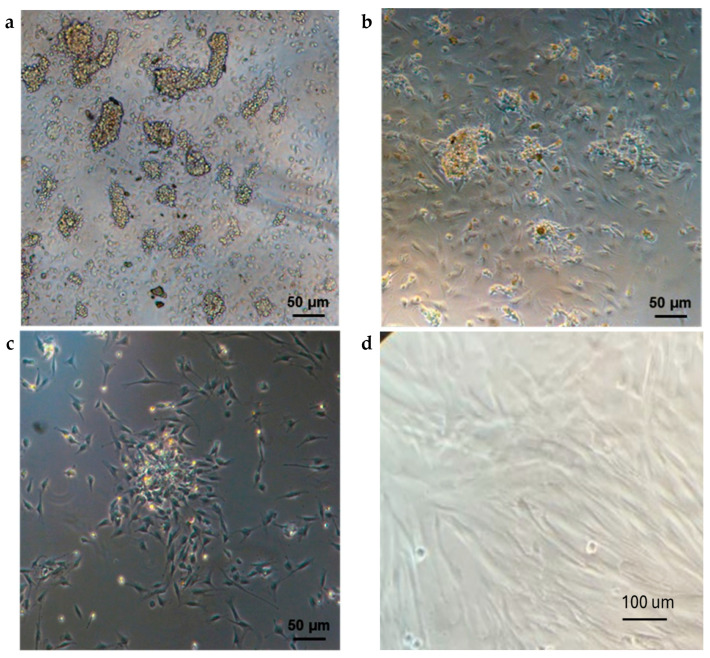
Morphological features of dental pulp stem cells (DPSCs). (**a**) One day after seeding; (**b**) After three days from seeding; (**c**) After the first passaging step; (**d**) DPSCs at Passage 2 (p2).
